# Supplementary material for: Social implications of coexistence of CAVs and human drivers in the context of route choice
Source: Sci Rep. 2025 Feb 25;15:6768. doi: 10.1038/s41598-025-90783-w (PMC11862007; doi:10.1038/s41598-025-90783-w)
Supplement: Supplementary file 1 — Supplementary Information. [file 41598_2025_90783_MOESM1_ESM.pdf]

Supplementary information to paper Social implications of coexistence of CAVs and human drivers in the context of route choice

Grzegorz Jamróz*[1], Ahmet Onur Akman[2], Anastasia Psarou[2], Zoltán Györgi Varga[2] , and Rafał Kucharski[1]

[1]Faculty of Mathematics and Computer Science, Jagiellonian University, Kraków, Poland
[2]Jagiellonian University, Doctoral School of Exact and Natural Sciences

1 Appendix

In this supplementary file we discuss various aspects of the research which motivated the main paper, which, however do not form part of the main exposition. We include also certain additional results and simulations, which either for space or significance reasons have been omitted in the main paper, which however provide a deeper understanding of the results and motivations.

The file is organized as follows. In Sections 1.1 and 1.2 we answer the following fundamental question:

In which settings can we reasonably evaluate the impact of sudden introduction of CAVs?

We propose, namely, to evaluate the impact of CAVs only in settings in which HDV choices stabilize. For this purpose, we conduct exhaustive experiments supporting our selection of the ϵ-Gumbel model.

In Section 1.3 we present exemplary results of simulations upon which the figures in the main text are based, presented however as day(time)-dependent plots. These time-dependent plots are included for completeness, as in the main text only a synthesis of results is included and no depiction of the dynamic process which evolves over the horizon of 400 days. Crucially, the process generally stabilizes, justifying the presenting of averages of means in the main text. Nonetheless, sometimes the process oscillates, which results in spread probability distributions as in Fig. 3 in the main text. Additionally, we provide and discuss simulations with Gumbel-distributed error term replaced by normally-distributed error terms and observe that the results are almost indistinguishable. In Section 1.4 we show that the core findings of the paper are very robust and reproducible, by repeating the experiments 10 times and conducting appropriate statistical tests. In Section 1.5 we discuss the impact of perception bias of human populations when dealing with fleets of CAVs which apply strategies other than selfish (which we consider most relevant, and which was the only one included in the main text). Finally, Section 2 contains a supplementary table presenting the parameters used in simulations presented in this appendix as well as all supplementary figures.

1.1 Stabilization in HDV-only systems

We present the results of a systematic experimental (in silico) study of equilibrium properties in the case of two routes. The default parameters as well as their alternative values are given in Supplementary Table 1. As logit models are the most important models accounting for human choice in transportation settings we set the default *Model Type* to 'Logit'. The *Dispersion Parameter* is by default $\gamma = 1/\beta = 0.2$. The 'Free Flow' *Initial Knowledge* seems to be reasonable, see Methods, however we also tested optimistic (0 min) and pessimistic (25 min) initial travel time estimates. Given the *Initial Knowledge*, we set 'Random' as the default *Initial Choice*, however 'ArgMin' also can be used. Our experiments assume that agents learn from experience only, however we compare this to learning from full knowledge of the travel times. The number of HDVs is fixed at 1000, which corresponds to moderate congestion. Finally, the learning rate is typically 0.2 [1] and exploration rate 0.1.

In every experiment, we vary two parameters and display the travel times on routes A and B. We conducted the following simulations and arrived at the following conclusions:

1. The impact of learning rate and dispersion in the logit model (Suppl. Fig. 1). We conclude that the default parameters $\gamma = 0.2$, $\alpha = 0.2$ result in the system stabilizing (with travel times on both routes substantially different). However, for certain other combinations of parameters the system either exhibits oscillations or fails to converge to a state similar to SUE.

2. The effect of oscillations is particularly pronounced in the full information case. Suppl. Fig. 2 demonstrates

this effect, which was theoretically studied in [2]. We note that in the full information case equation (6) is replaced by

$$T_r(i) \leftarrow (1-\alpha)T_r(i) + \alpha t_r(i)$$

for $r \in \{A, B\}$, i.e. estimates on *both* the used and unused routes are updated.

3. By default we assume that the initial knowledge of every agent corresponds to free flow times. What might happen if this assumption is altered is shown in Suppl. Fig. 3. Interestingly, pessimistic initial knowledge results in agents not learning at all for high γ. However, both FreeFlow and Optimistic cases seem to converge to a situation when the agents have learnt the equilibrated travel times.

4. We generally assume that the initial choice of the route is random. Suppl. Fig. 4 demonstrates that the choice Argmin (selecting the route with a better initial estimate) leads to the same results.

5. Suppl. Fig. 5 shows that the Logit and ϵ-Gumbel models (note we use the names ϵ-Gumbel and GumbelEps interchangeably) exhibit similar behaviour in general. The Gumbel model, on the other hand, sometimes converges to a state where the equilibrium travel times remain 'unlearned'. The Gumbel model differs from the default ϵ-Gumbel model by replacing equation (8) by

$$r(i) = \arg\min_{r\in\{A,B\}} U_r(i), \tag{A1}$$

i.e. by removing the extra random exploration. The Logit model, in turn, differs from the default ϵ-Gumbel model by replacing (8) with (A1) and by sampling $\varepsilon_r(i)$ in (7) from $\mathit{Gumbel}(\mu, \beta)$ every day anew, independently for every agent i and route r (instead of keeping them fixed and sampled once on day 1). Equivalently [14], equation (8) can be replaced by choosing A with probability given by the logit formula

$$P_A = \frac{\exp(-T_A(i)/\beta)}{\exp(-T_A(i)/\beta) + \exp(-T_B(i)/\beta)}, \tag{A2}$$

and $P_B = 1 - P_A$.

This standard logit model has a different interpretation to ϵ-Gumbel. Namely, the heterogeneity of choices is not caused by fixed different tastes which on average produce logit proportions on alternatives, but rather by random everyday fluctuations of driving conditions or agent preferences.

6. Suppl. Fig. 6 shows the behaviour of ϵ-Greedy Model for various values of parameters α and knowledge. The ϵ-Greedy model is the limit of ϵ-Gumbel for $1/\gamma = \beta \to 0$. More precisely, we replace equation (8) by

$$r(i) = \begin{cases} \arg\min_{r\in\{A,B\}} T_r(i) & \text{with probability } 1-\epsilon, \\ \text{uniformly random} & \text{with probability } \epsilon. \end{cases}$$

It can be seen that wild oscillations prevail in the case of full knowledge and some instability can be observed for learning from experience only. We conclude that if learning never stops, ϵ-Greedy choice might become unstable.

7. This inherent instability is shown in Suppl. Fig. 7. Note that for $\alpha = 0.2$ and $\epsilon = 0.1$ what appeared as a stable pattern during the first 200 days turned out to be unstable in a longer-term horizon. We conclude that in order to consider greedy choice as a baseline, we need to either halt human learning at some point or be very cautious in order not to mistake the instability of the model for the impact of CAVs. Because of this instability as well as its unrealistic interpretation of humans with no biases whatsoever, we do not consider this model in further experiments.

1.2 Conclusion

We select the multi-agent ϵ-Gumbel model with default parameters listed in Table 3 as our benchmark motivated as follows. On the one hand, it allows for certain diversity among agents and renders the agents' decisions deterministic up to ϵ-size exploration (note that ε_r determining human tastes are sampled only once, while exploration with probability ϵ may occur every day), which seems to be a reasonable tradeoff between simplicity and realisticity. On the other hand, it stabilizes across a wide range of reasonable parameters. Importantly, the ϵ-Gumbel model and the more common standard Logit model result in similar proportions of choices as confirmed by our experiments. However, as motivated above, we prefer ϵ-Gumbel as it seems to be a simple model with sounder behavioural underpinnings.

1.3 Time-dependent plots of CAV-HDV interaction

In Suppl. Fig. 8 and 9 we present the time dependent plots for a selection of models and various parameters upon which the plots in the main text are based (for Model = GumbelEps). In particular, we see that the logit model and the ϵ-Gumbel model behave similarly with a slight difference in variance of vehicle counts or travel times. Moreover, for the default parameters the flows and travel times eventually stabilize after Day-M (day 200). Nonetheless, for certain CAV shares, the malicious and disruptive strategies can be executed most efficiently keeping the system permanently

out of equilibrium by introducing oscillations visible in the plots.

Suppl. Fig. 10 shows the results of simulations with error terms which are drawn from the normal distribution with mean 0 and variance equal to the variance of the Gumbel distribution used to produce Fig. 9. Comparing the two figures, one can notice that they are virtually indistinguishable, which confirms that the normally-distributed and Gumbel-distributed error terms result in similar outcomes.

1.4 Reproducibility and statistical significance

For parameters used in Fig. 3 we reran the experiments independently 10 times obtaining satisfactory reproducibility, Suppl. Fig. 11. In particular, for the combinations of parameters reported in Fig. 2 we obtained statistical significance of the difference between mean HDV travel time averaged over days $101-200$ (HDVb) and mean HDV travel time averaged over days $301-400$ (HDV), expressed by $pHDV$, as well as between mean HDV travel time averaged over days $101-200$ (HDVb) and mean CAV travel time averaged over days $301-400$ (CAV), expressed by $pCAV$. We used the paired two-sided t-test to test the null hypothesis of equality of the respective averaged mean travel times and obtained in every case reported in the synthesis Fig. 2 p-values less than 0.001.

1.5 Impact of perception bias (spread of human preferences) for optimization strategies other than selfish

Suppl. Fig. 12 shows the positive and negative consequences of introduction of CAVs for different spread of human preferences for strategies other than the most relevant selfish strategy discussed in Fig. 7. The Social strategy exhibits effects similar to the selfish strategy. On the other hand, the impact of the altruistic strategy is much deeper and results in huge costs for CAVs(i.e. effect of changing to CAV $\ll 1$), especially for lower biases and high CAV shares as well as considerable gains for HDVs (Effect of remaining HDV $\gg 1$). The malicious strategy should be assessed mainly via the negative Effect of remaining HDV. This effect is most pronounced for high biases of HDVs. Interestingly, for biases large enough, the malicious strategy is efficient (i.e. harmful to HDV performance) at the level of travel times (Effect of remaining HDV < 1), however it fails at the level of utility (Perceived effect of remaining HDV > 1). The cost of executing the malicious strategy is rather high for large CAV shares or very large HDV biases. For smaller shares and small bias the Effect of changing to CAV is > 1 which means that the malicious strategy is in fact beneficial to CAVs. Finally, the disruptive strategy exhibits effects similar to the malicious one, however with reduced scale of the effects and reduced cost (or even benefit for CAVs) for high HDV perception bias.

1.6 Impact of explicit HDV $\leftrightarrow$ CAV switching dynamics for fleet targets other than selfish

Suppl. Fig. 13 presents the mean travel times of HDVs before the introduction of CAVs (days 101-200) and of HDVs and CAVs after the introduction of CAVs (days 301-400) when agents are freely allowed to switch (arbitrarily many times) between HDVs and CAVs for strategies other than selfish (which is presented in Fig. 10).

References

[1] Bogers, E. A. I., Bierlaire, M. and Hoogendoorn, S. P. (2007). Modeling Learning in Route Choice. Transportation Research Record, 2014(1), 1-8. https://doi.org/10.3141/2014-01

[2] Horowitz, Joel L. (1984). The stability of stochastic equilibrium in a two-link transportation network. Transportation Research Part B: Methodological, Elsevier, vol. 18(1), pages 13-28, February.

2 Supplementary Tables and Figures

Supplementary Table 1: HDV parameter values used to study the properties of equilibria.

Parameter	Default Value	Alternative Values
Model Type	Logit	Gumbel, ϵ-Gumbel, ϵ-Greedy
Model Parameter γ (Logit parameter by default)	0.2	0.01 – 3.0
Initial Knowledge	Free Flow	Optimistic, Pessimistic
Initial Choice	Random	ArgMin
Learning From Experience Only	True	False
HDV number	1000	–
Learning rate of HDVs (α)	0.2	0.1 – 1.0
Exploration rate of HDVs (ϵ)	0.1	0.01 – 0.5

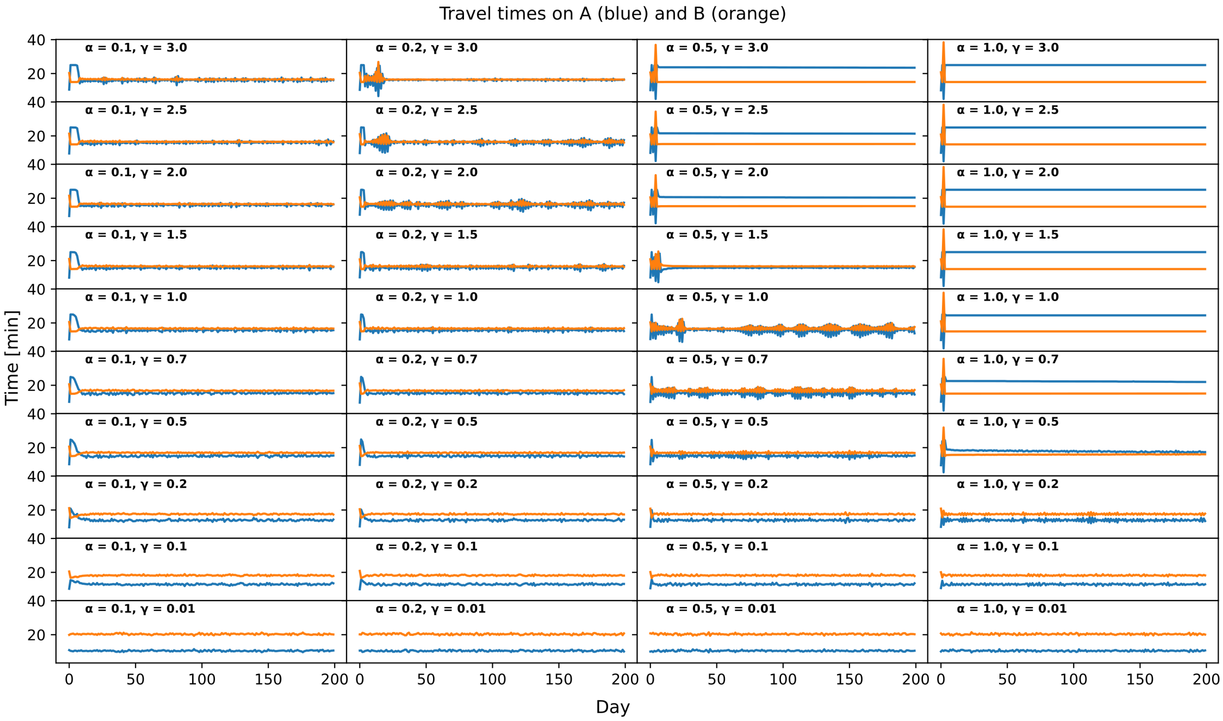

Supplementary Figure 1: Travel time in the logit model for different learning rates and logit parameters. One can observe substantial oscillation for certain combinations of parameters, especially for higher values of γ with $\alpha = 0.2$ and γ in the range $0.5 - 1.0$ for $\alpha = 0.5$. These oscillatory solutions are hardly realistic and it seems that testing CAV introduction impact in these ranges might be problematic.

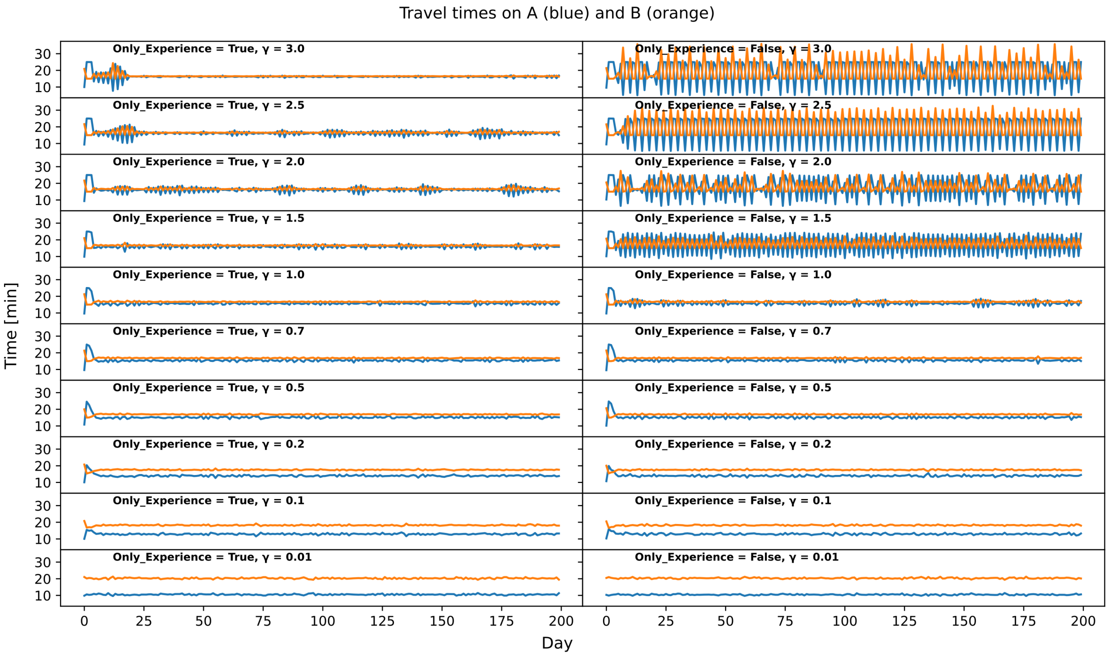

Supplementary Figure 2: Oscillations in the logit model for different dispersions in the case of learning from experience only and learning from full information. Here, $\alpha = 0.2$ by default. It can be observed that when agents have access to full information about travel times, the oscillations are much more likely and have increased amplitude. Therefore using models with learning from own experience only seems to be less methodologically problematic.

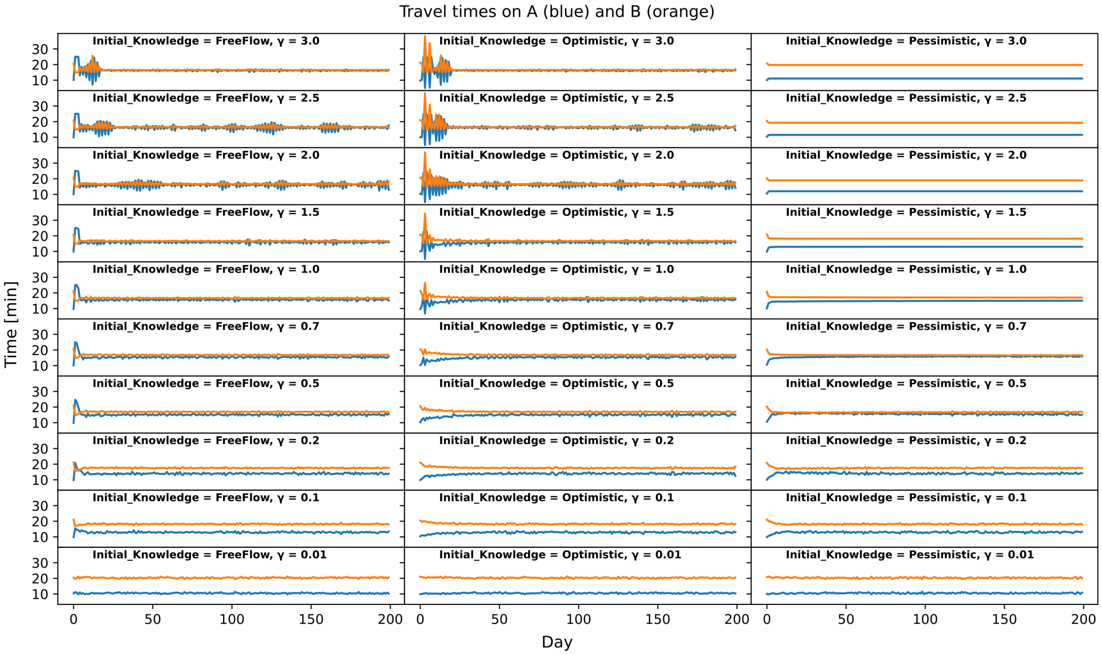

Supplementary Figure 3: Travel time in the logit model for different Initial Knowledge and logit parameters. It can be seen that for initial knowledge of the agents which corresponds to FreeFlow travel times or Optimistic initial estimates of travel times equal to 0.0 the system converges to a state fluctuating around certain equilibrium values. This is in general not the case, however, for Pessimistic initial knowlege which seems to be unsuitable for our simulations. Note, however, that for the default $\gamma = 0.2$ even the Pessimistic case converges.

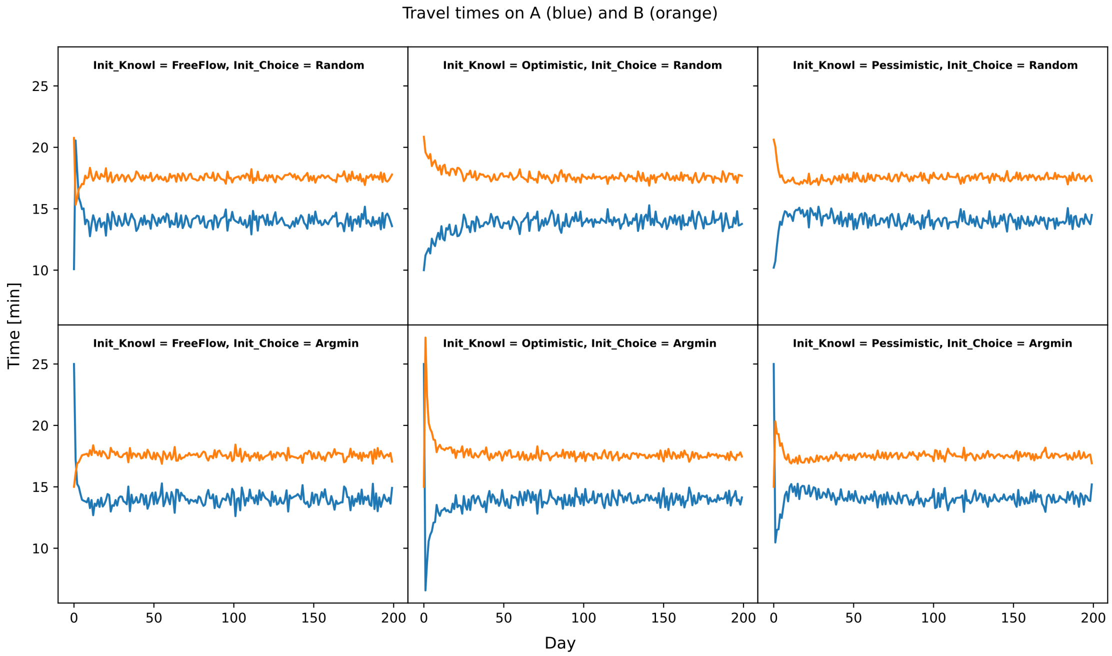

Supplementary Figure 4: Travel time in the logit model for different Initial Knowledge and Initial Choice modes. The initial choice – random or argmin seem to have little effect on the long-term behaviour of the simulation for the default parameter values.

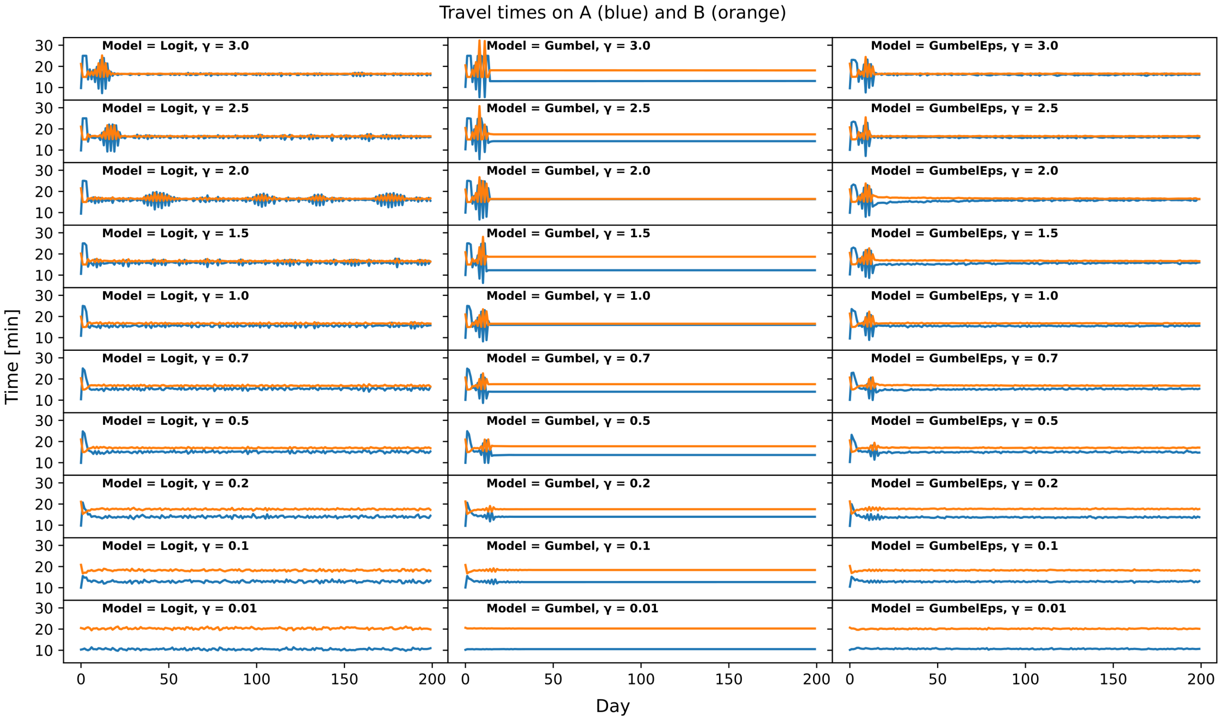

Supplementary Figure 5: Travel time for different dispersions γ in different logit-type models. One can observe that both Logit and GumbelEps seem to converge nicely to fluctuations around the stable states. The Gumbel model, however, exhibits a different behaviour, with agents often 'stuck' in wrong choices, because of lack of explicit exploration.

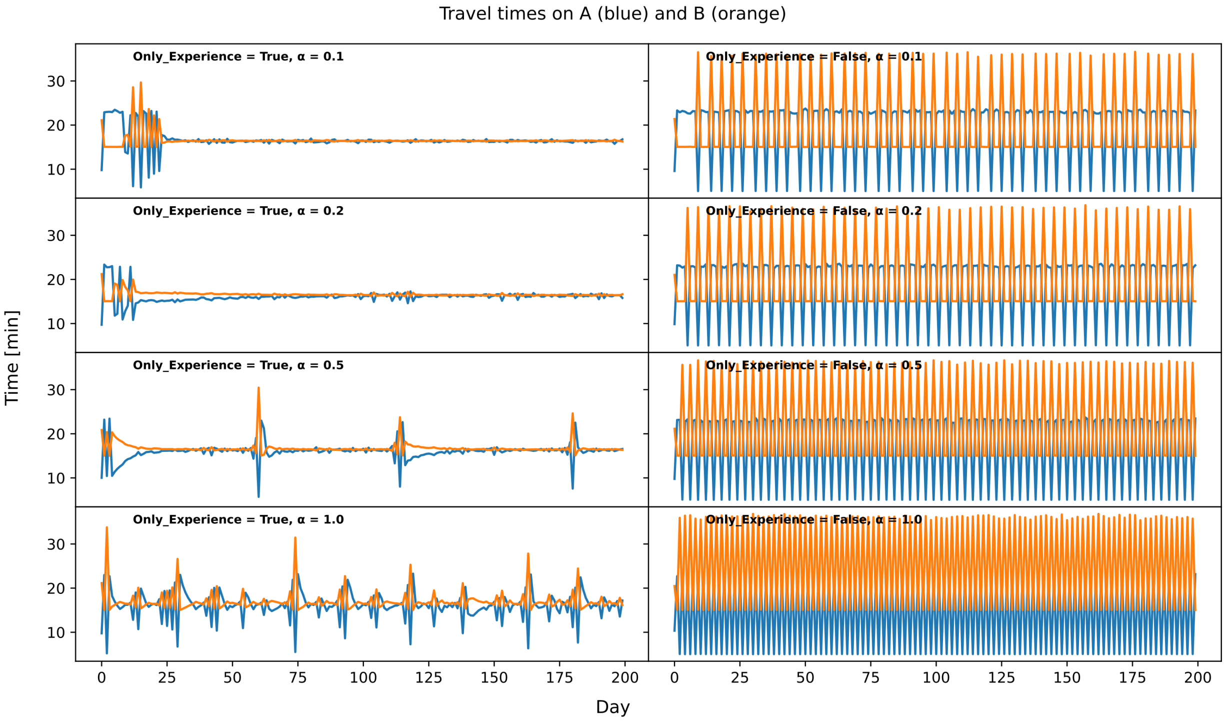

Supplementary Figure 6: Travel time in the ϵ-greedy model for different learning rates and experience. It seems clear that this model yields very unstable results and does not seem appropriate for studying the impact of CAVs.

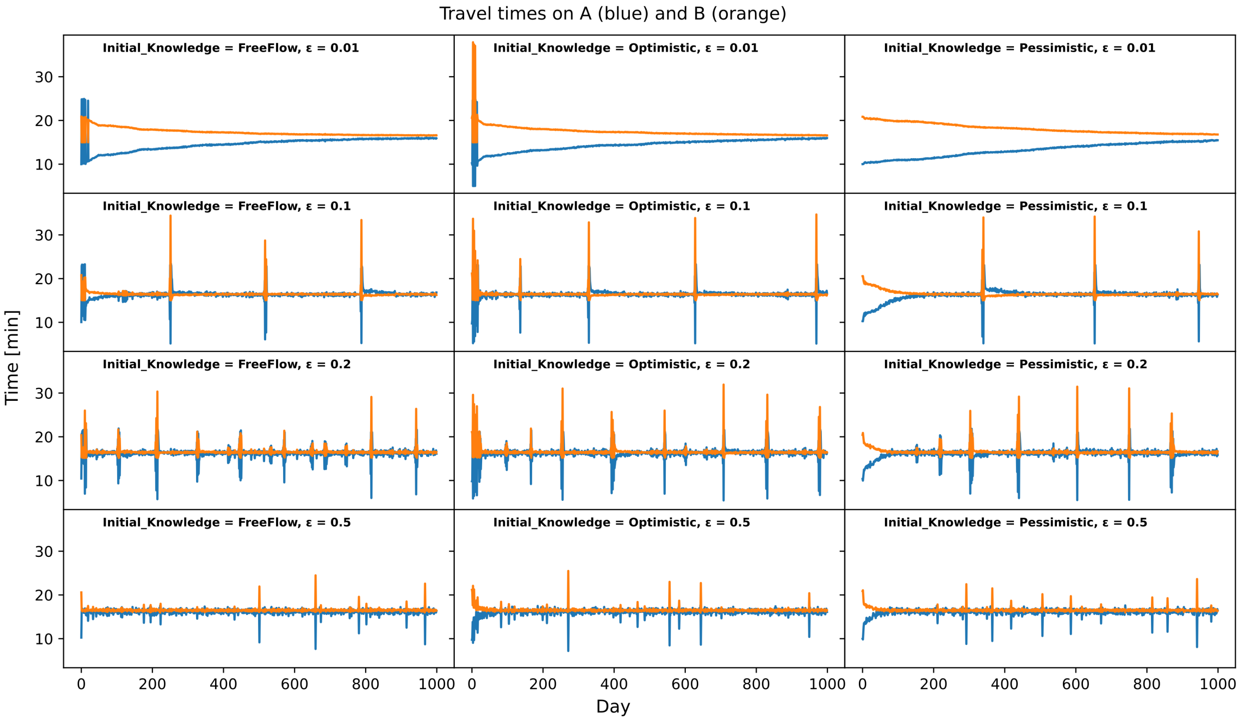

Supplementary Figure 7: Long term behaviour of the system with ϵ-greedy human choice for various exploration rates and initial knowledge. The system is prone to spikes once the travel times have equilibrated. Therefore, using it to study the impact of CAVs is problematic.

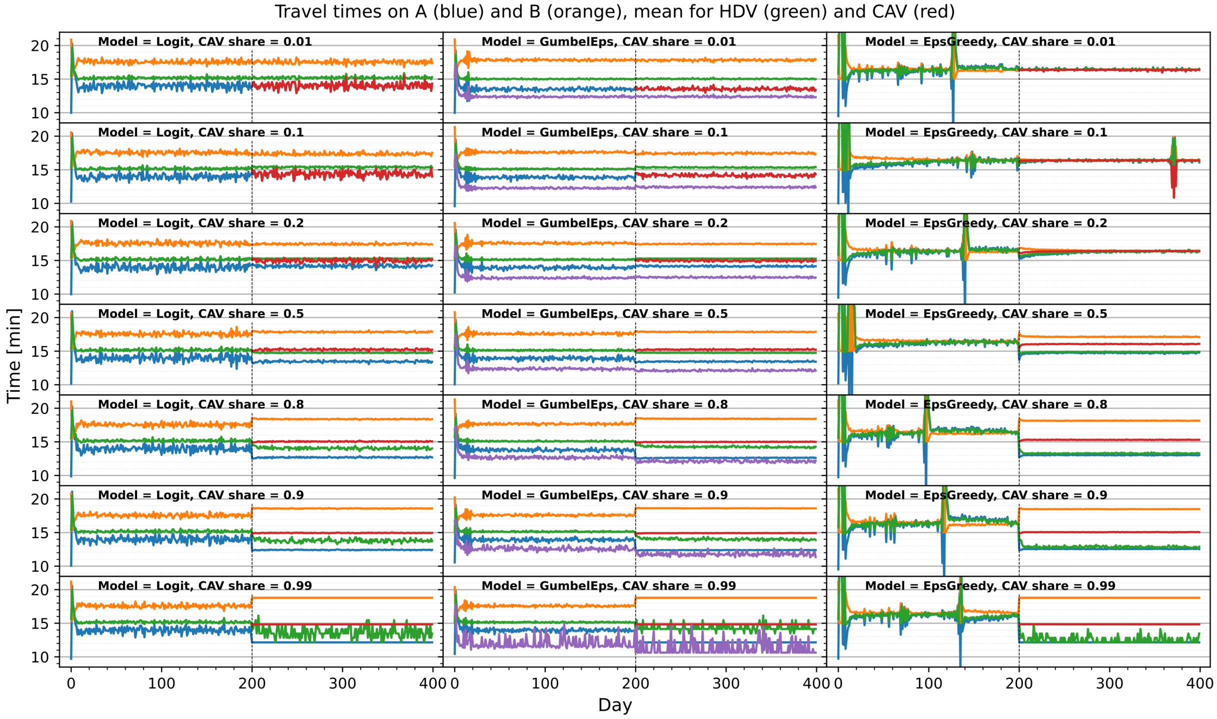

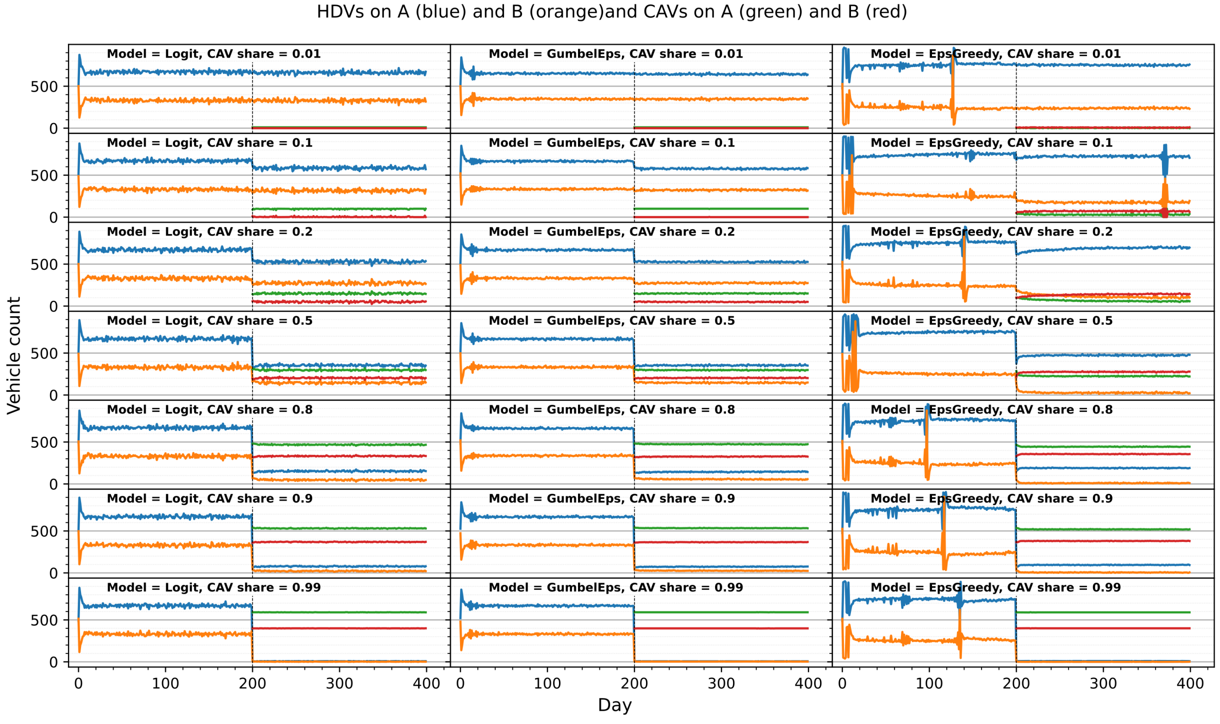

Supplementary Figure 8: Travel times and vehicle counts for different logit-type human behaviour models and CAV shares and selfish strategy. Purple – mean perceived travel time. One can observe spikes in the EpsGreedy model, which are problematic for studying coexistence of CAVs and HDVs. On the other hand, the graphs for Logit and GumbelEps models are similar, however in the case of GumbelEps, additionally the mean perceived travel time of HDVs (i.e. utility, which is the sum of travel times and fixed tastes, eq. (3)) is plotted.

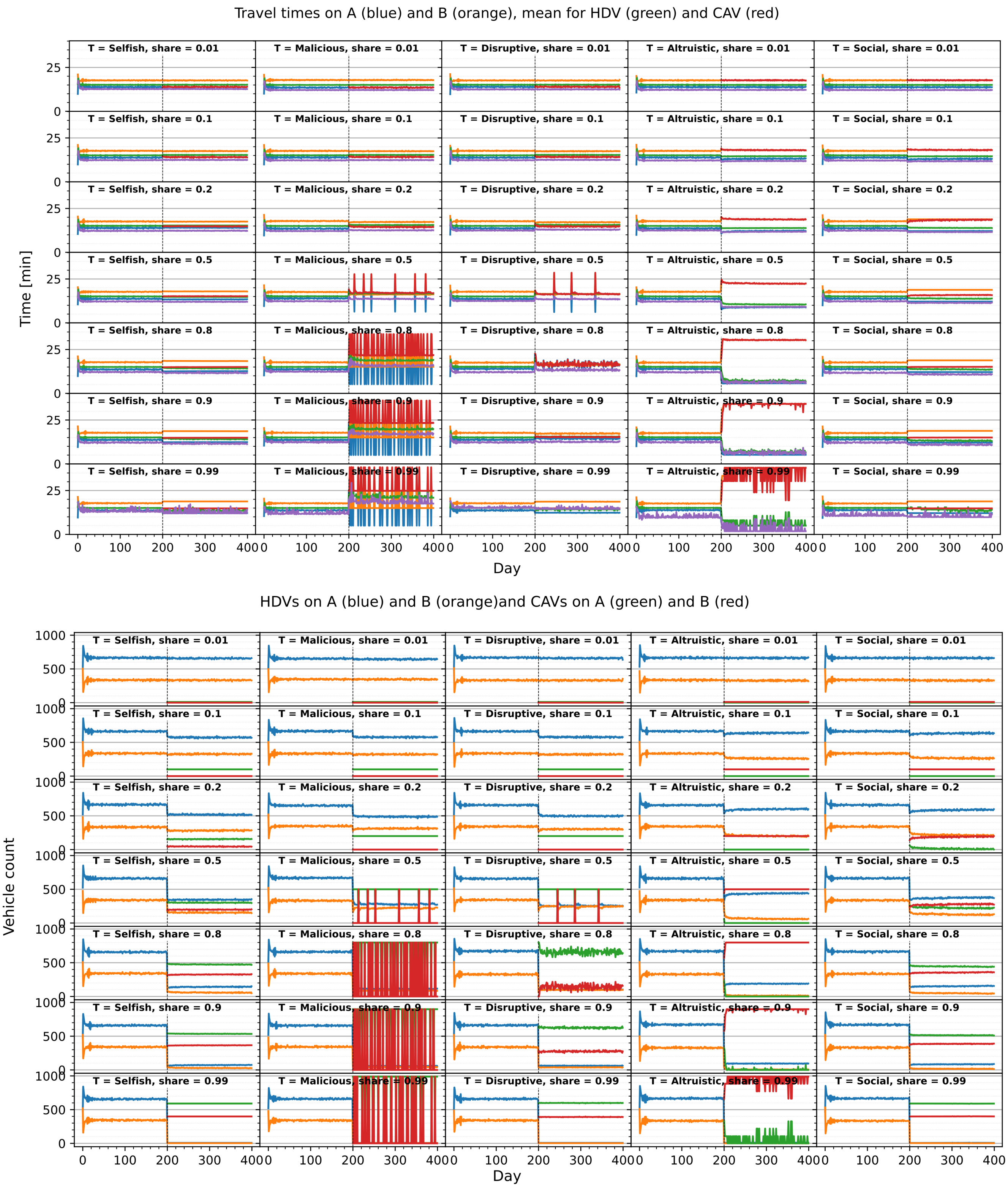

Supplementary Figure 9: Travel times and vehicle numbers for different CAV optimization targets and CAV shares in the ϵ-Gumbel model beyond the M-day 200 on which CAVs are introduced. We observe that the vehicle counts and travel times stabilize before day 200. Afterwards, they usually stabilize towards day 400 as well, however the malicious and disruptive strategies of CAVs often involve keeping the system out of balance, which can be seen in the plots. Purple – mean perceived travel time.

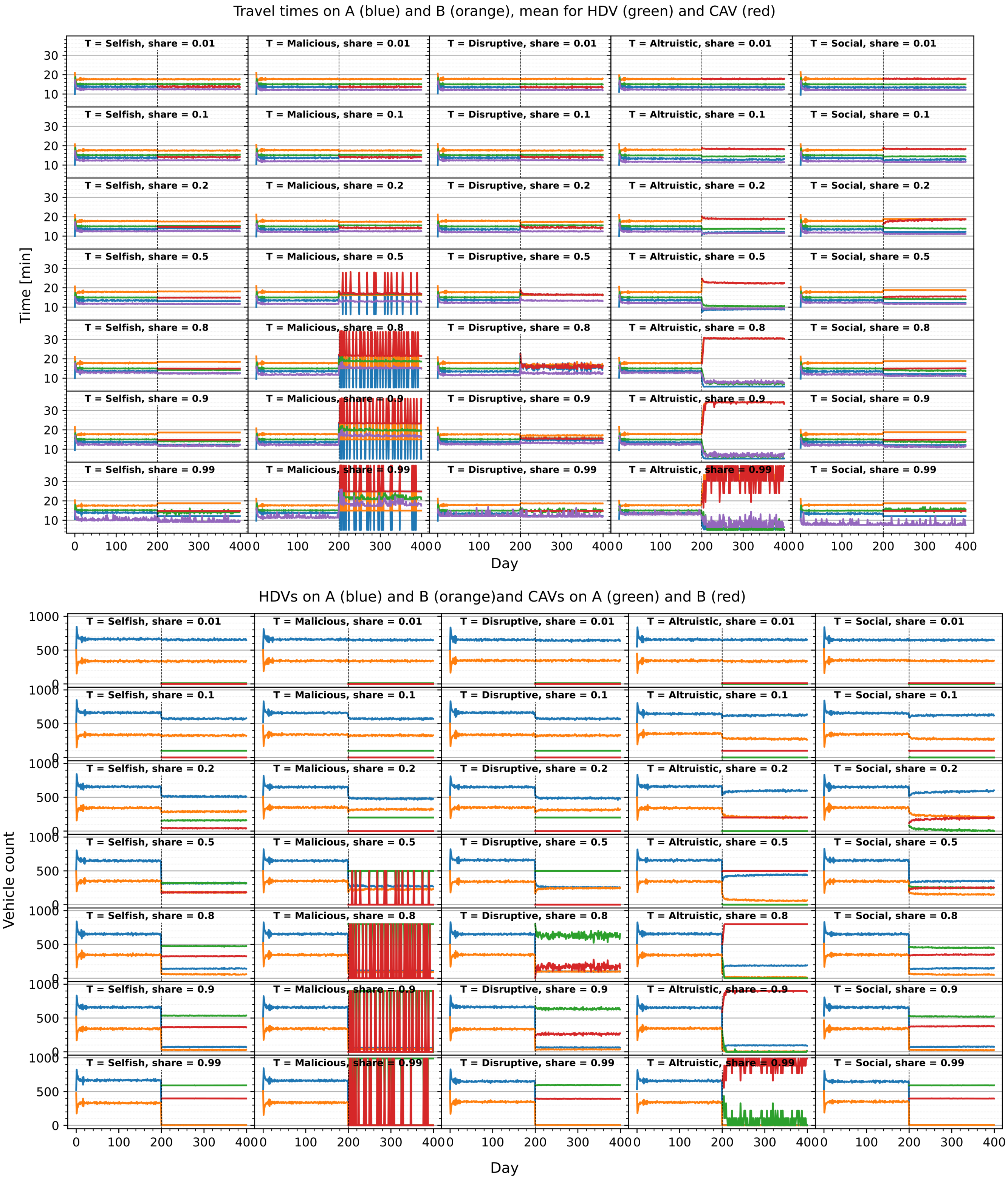

Supplementary Figure 10: Mean Travel times and vehicle numbers for different CAV optimization targets and CAV shares in the ϵ-Normal model, i.e. the model with error terms in eq. (3) drawn from the Normal distribution. Purple – mean perceived travel time. Comparison to Suppl. Fig. 9 reveals no significant differences and confirms that the ϵ-Gumbel and ϵ-Normal models are interchangeable.

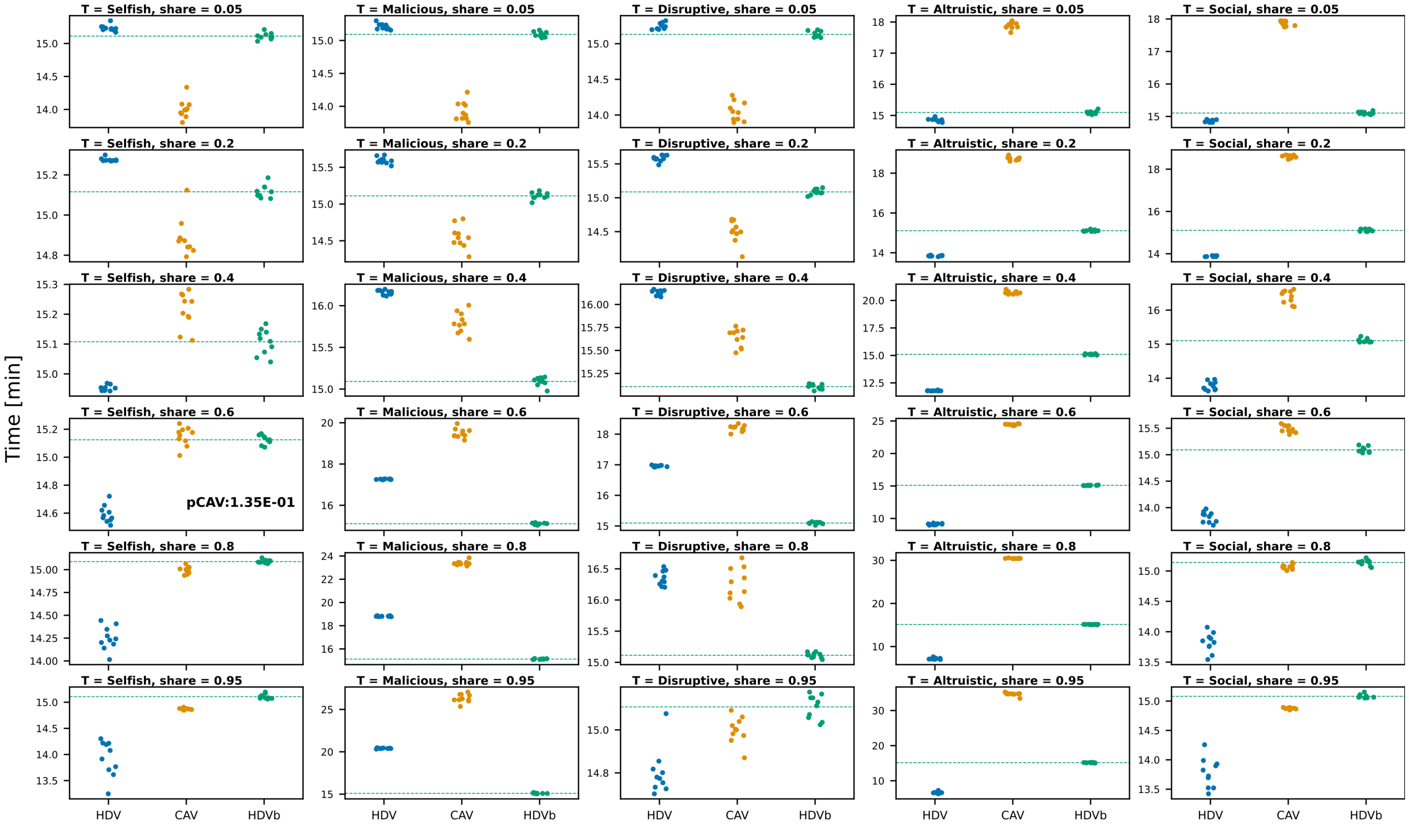

Supplementary Figure 11: Mean travel times of HDVs averaged over days $301 - 400$ (HDV), mean travel times of CAVs averaged over days $301 - 400$ (CAV) and mean travel times of HDVs averaged over days $101 - 200$ (HDVb) obtained in 10 independent experiments for different strategies and CAV shares. The green dotted line is the mean of the sample of HDVb. The mean of HDV vs. mean of HDVb as well as mean of CAV vs. mean of HDVb are all statistically different with $p < 0.001$ (two-tailed paired t-test) except for the selfish strategy for CAV share 0.6. Only the p-values larger than 0.001 are displayed. $pCAV$ – p-value for the hypothesis of equality of CAV and HDVb. $pHDV$ – p-value for the hypothesis of equality of HDV and HDVb.

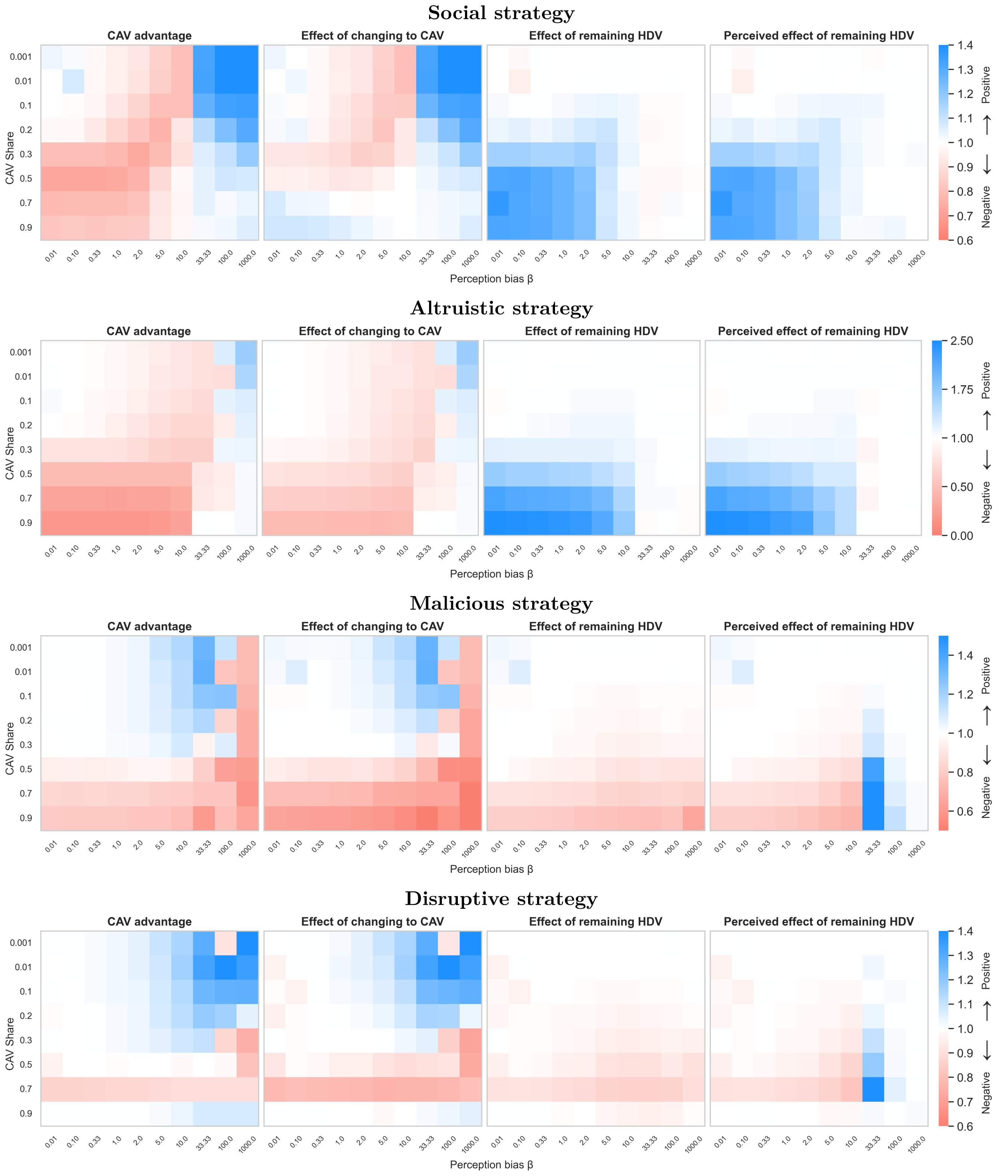

Supplementary Figure 12: Effect of a given share of vehicles mutating to CAVs for different perception biases and strategies other than the default selfish strategy.

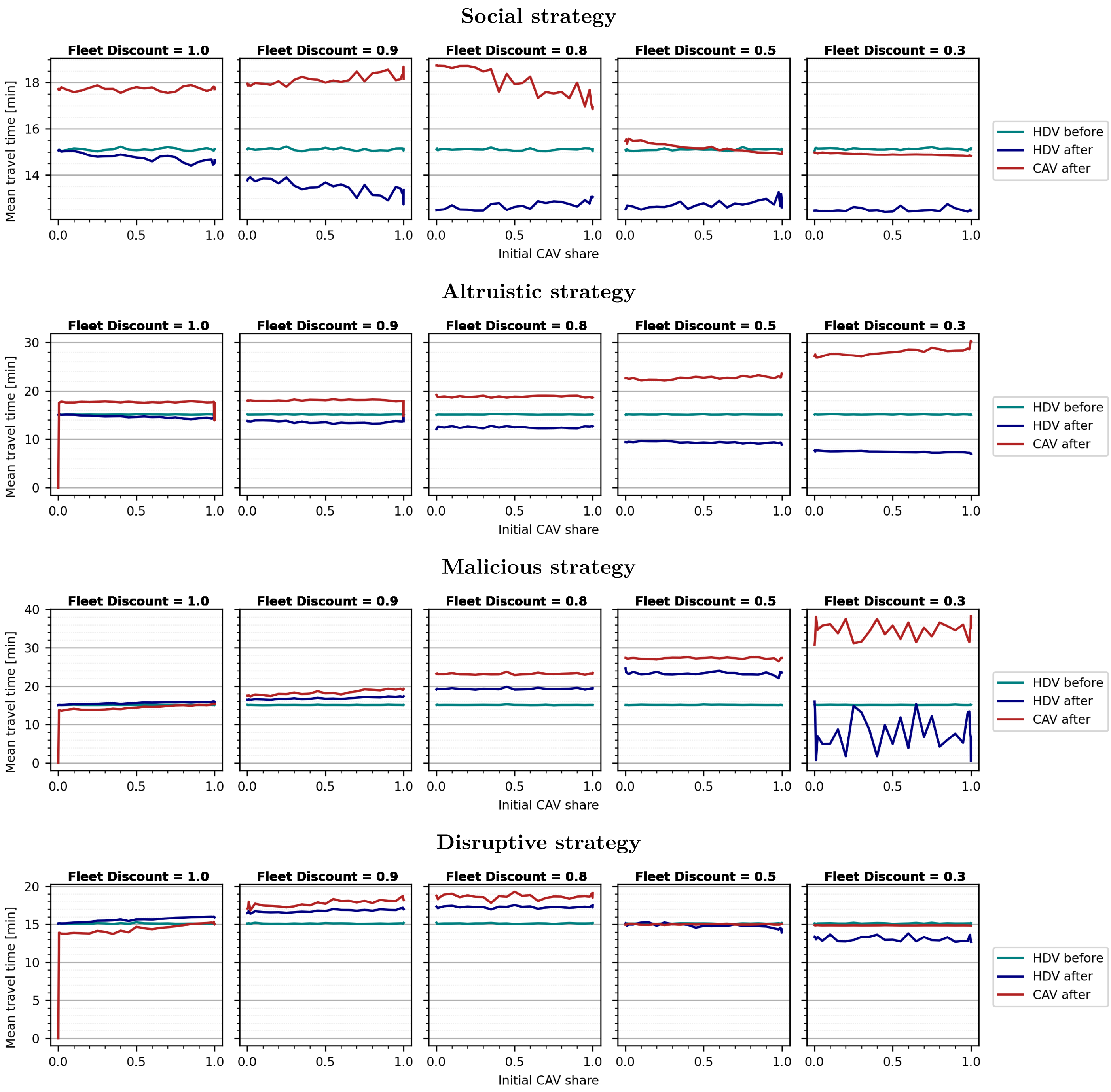

Supplementary Figure 13: Mean travel times of HDVs averaged over days 101-200 (HDV before) or 301-400 (HDV after) and of CAVs averaged over days 301-400 (CAV after) for fleet strategies other than selfish and all agents allowed to switch between HDV and CAV.
